# Morpho-functional gastric pre-and post-operative changes in elderly patients undergoing laparoscopic cholecystectomy for gallstone related disease

**DOI:** 10.1186/1471-2482-12-S1-S5

**Published:** 2012-11-15

**Authors:** Giovanni Aprea, Alfonso Canfora, Antonio Ferronetti, Antonio Giugliano, Francesco Guida, Antonio Braun, Melania Battaglini Ciciriello, Federica Tovecci, Giovanni Mastrobuoni, Fabrizio Cardin, Bruno Amato

**Affiliations:** 1 Department of General, Geriatric, Oncologic Surgery and Advanced Technologies, University “Federico II” of Naples, Via Pansini, 5 - 80131 – Naples, Italy; 2 Department of Surgical and Gastroenterological Sciences, Padova University Hospital, Italy Via Giustiniani n.2, 35126 Padova, Italy

## Abstract

**Background:**

Cholecystectomy, gold standard treatment for gallbladder lithiasis, is closely associated with increased bile reflux into the stomach as amply demonstrated by experimental studies. The high prevalence of gallstones in the population and the consequent widespread use of surgical removal of the gallbladder require an assessment of the relationship between cholecystectomy and gastric mucosal disorders.

Morphological evaluations performed on serial pre and post – surgical biopsies have provided new acquisitions about gastric damage induced by bile in the organ.

**Methods:**

62 elderly patients with gallstone related disease were recruited in a 30 months period. All patients were subjected to the most appropriate treatment (Laparoscopic cholecystectomy). The subjects had a pre-surgical evaluation with:

• dyspeptic symptoms questionnaire,

• gastric endoscopy with body, antrum, and fundus random biopsies,

• histo-pathological analysis of samples and elaboration of bile reflux index (BRI).

The same evaluation was repeated at a 6 months follow-up.

**Results:**

In our series the duodeno-gastric reflux and the consensual biliary gastritis, assessed histologically with the BRI, was found in 58% of the patients after 6 months from cholecystectomy. The demonstrated bile reflux had no effect on H. pylori’s gastric colonization nor on the induction of gastric precancerous lesions.

**Conclusions:**

Cholecystectomy, gold standard treatment for gallstone-related diseases, is practiced in a high percentage of patients with this condition. Such procedure, considered by many harmless, was, in our study, associated with a significant risk of developing biliary gastritis after 6 months during the postoperative period.

## Background

A thorough review of literature has shown that cholecystectomy is accompanied, in the years following surgery, by an increase in duodenal-gastric reflux (DGR) [1-3], however, there is only partial data on the incidence of bile reflux gastritis in patients undergoing cholecystectomy [4-7].

Numerous studies have also shown an association between cholecystectomy and gastric cancer [8-12]; the increased bile reflux may be a determining factor, if this risk was confirmed.

It is common, in patients who undergo cholecystectomy, to observe a persistence of upper abdominal symptoms often labeled as post-cholecystectomy syndrome [3,13-17]; these symptoms are likely related to a post-surgical duodenal-gastric reflux.

There are still conflicting reports on the possible effects of duodenal-gastric reflux on Helicobacter pylori’s gastric infection.

We therefore decided to perform a prospective study on elderly patients which refer to our general surgery department in order to evaluate whether cholecystectomy increases the risk of gastritis, induces the onset of dyspeptic symptoms, alters the incidence of *H. pylori* infection or increases the risk of gastric cancer.

## Methods

The primary objective of our study was to evaluate the incidence of postoperative biliary gastritis through the prospective evaluation of patients with symptomatic gallbladder lithiasis treated with laparoscopic cholecystectomy.

Among the secondary objectives were:

- Assessment of changes in prevalence of *H. pylori* gastritis resulting from the eventual bile reflux gastritis

- Assessment of the presence of lesions, in the follow-up, considered as risk factors for gastric cancer

The study was carried out with the following modalities:

1) recruitment of elderly patients (over 70) with symptomatic gallstones and ultrasound documentation of the stones for which there was indication for cholecystectomy and who gave informed consent for the study participation.

2) exclusion of all patients exposed to other risk factors such as NSAIDs or alcohol who are able to determine gastric symptoms and / or reactive gastritis.

3) pre-surgical evaluation:

• dyspeptic symptoms questionnaire subministration (Table 1),

• gastric endoscopy in order to assess the absence of macroscopically visible lesions and to provide multiple biopsies of the gastric antrum, body and fundus,

• histo-pathological analysis of samples and elaboration of bile reflux index (BRI). This index is elaborated grading the following four histological parameters on a scale from 0 to 3: lamina propria edema (Oed), chronic inflammation (CI), intestinal metaplasia (IM), *Helicobacter pylori* colonization density (Hp). Subsequently the following formula is applied to obtain the BRI:(7xOed) + (3xIM) + (4xCI) - (6xHp).

**Table 1:** Dyspeptic symptoms questionnaire

Symptom	Pre-operative score(0-3)	Post-operative score(0-3)
Epigastric pain		

Nausea		

Bilious vomiting		

Upper abdominal quadrant swelling		

Post-prandial fullness		

Heartburn		

Frequent belching		

A value of BRI equal to or greater than 14 is indicative of reflux gastritis and if used as a single diagnostic investigation for pathological DGR has a sensitivity of 70% and a specificity of '85% to detect a bile level > 1.00 mmol / L in the stomach (upper limit of physiological bile reflux) [18-20]. Patients that may be positive for *H. pylori* should not perform eradication therapy at least until the first follow-up at 6 months.

4) laparoscopic cholecystectomy.

5) clinical reassessment of patients at 6 months postoperatively with the dyspeptic symptoms questionnaire and new endoscopy for BRI score evaluation.

6) Comparison of clinical and histopathological data obtained during pre-surgical phase and during follow-up at 6 months.

The study has provided so far, since January 2010, the enrollment of 62 patients. Of these, 31 completed the follow-up to 6 months, 19 were lost at follow-up, 12 patients have yet to complete the follow-up. Nineteen of 62 patients (30.64%) did not return for the post-operative follow-up ,maybe for the scant willingness to undergo an invasive follow-up endoscopy, especially if the purpose is the finding of a bile reflux gastritis, a condition that can occur without symptoms and who’s long term risks are unknown. Patients who did not undergo the postoperative examination were excluded from the study.

## Results and discussion

Of the 31 patients who completed follow-up (50%), 13 were men and 18 women. The age was between 70 and 85 years with an overall mean age of 74.86. The age range in the male group was between 71 and 85 years with a mean age of 75.09 years. In female subjects the minimum age was 70 years and the maximum age was 80 years, with an average of 74.70 years.

The clinical evaluation showed, in pre-operative phase, in all cases, the presence of dyspeptic symptoms. However we must remember that symptoms such as upper abdominal pain, nausea and bilious vomiting are also attributable to episodes of "biliary colic" related to the gallstone disease.

However, it results difficult to attach any dyspeptic symptom found to a possible pathological DGR present in the pre-surgical phase and to its associated morphological changes cause in 23 of 31 examined subjects a coexisting *H. pylori* infection could have been responsible for similar symptoms.

The persistence in some subjects (13 of total 31 examined), after surgical removal of the gallbladder, of the previous symptoms could, on the other hand, be related to the onset of a pathological DGR and the associated morphological changes. However, resulting the *H.pylori* infection prevalence unchanged , it remains difficult, based on clinical observations, to attribute these symptoms solely to DGR. Final results would require assessments of groups of subjects negative for *Helicobacter pylori* infection that we have chosen not to follow exclusively to evaluate the in vivo effect of bile acid levels on the *H. pylori* infection in subjects after cholecystectomy.

Preoperative histo-pathological findings showed the following:

Of the 31 patients examined 23 were positive for *Helicobacter pylori* infection in pre-operative and in all these subjects the antrum was always affected alone or in the context of a pan-gastritis. The infection was associated with morphological features of a mild or moderate chronic gastritis.

In all studied subjects , even in *H. pylori* negative areas, chronic inflammatory changes were highlighted; such thing could be explained by an abnormal duodenal-gastric reflux related to the gallbladder exclusion operated by lithiasis. In 8 cases these modifications were such that, also pre-operatively, in one or more portions of the stomach, the BRI exceeded the threshold value of 14.

Since the calculation of the BRI gives a different weight to the various evaluated morphological entities and in particular requires the subtraction of the density of *Helicobacter pylori* colonization in order to detect the presence of chronic inflammation related to duodenal-gastric reflux, it is easy to understand that, although there is an antrum-body-fundus gradient for the damage caused by DGR, because the antrum is the most common site of bacterial colonization, the average BRI value calculated in this site is going to be lower than that of the body and of the fundus (Table 2).

**Table 2:** Pre-operative BRI values.

	BRI mean value	SD
**Antrum**	8.5	5.2

**Body**	10.69	7.06

**Fondus**	10.15	6.33

In the post-operative phase at 6 months there is an increase of the mean values of BRI in all patients and in all portions of the stomach, while the incidence of *Helicobacter pylori* results unchanged (Table 3 and 4).

**Table 3:** Post-operative BRI values.

	BRI mean value	SD
**Antrum**	15.82	7.83

**Body**	17.24	7.72

**Fondus**	16.93	7.48

**Table 4:** H. pylori positivity in pre e post-operative phase

	H. pylori -	H.pylori +
**Pre-operative**	8	23

**Post-operative**	8	23

As a result,the number of individuals with one or more locations with a BRI value > 14 increases from 8 subjects in the preoperative phase to 18 patients in the postoperative one.

This data was statistically tested in order to demonstrate the existence of an association between the surgical removal of the gallbladder and the occurrence of bile reflux gastritis. In first instance we organized a table for observed frequencies (Table 5). Using the χ2 test we compared the observed frequencies with the expected ones if the two conditions in question were independent .

**Table 5:** Observed frequencies

	BRI < 14	BRI > 14
**Pre-operative**	23	8

**Post-operative**	13	18

The value of the χ2 obtained from processing our data, with one degree of freedom, is 5.365 with a p value of 0.0205, and since the critical value of χ2 for one degree of freedom and with a probability of 5% is 3, 84 is possible to establish with this level of security the existence of an association between cholecystectomy and the onset of biliary gastritis.

In our sample there was no finding of intestinal metaplasia in either preoperative or postoperative phase. This result is consistent with the theory that intestinal metaplasia, although a precancerous lesion, requires a longer time (more than six months) to develop.

## Conclusions

**Figure 1:**
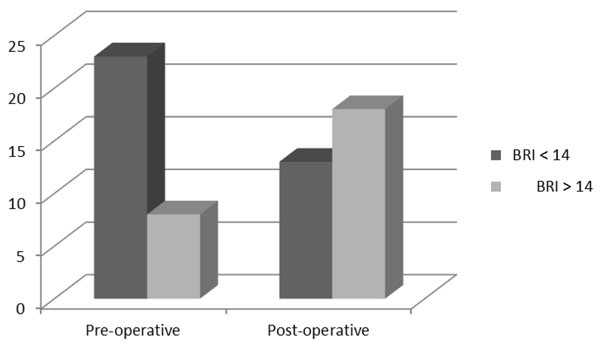
BRI positive patients in pre- and post-operative phase

Cholecystectomy, gold standard treatment for gallstone-related diseases, is practiced in a high percentage of patients with this condition. Such procedure, considered by many harmless, was, in our study, associated with a significant risk of developing biliary gastritis after 6 months during the postoperative period. This occurrence was found in our series in 58% of patients who underwent cholecystectomy (Fig. 1). However, the presence of symptoms in post-operative timing does not reflect the histological findings in these same patients: while, in fact, a positive histological BRI was found in 58% of patients after cholecystectomy, clinical symptoms were found in 41.9% of them .In addition these symptoms could also be related to the persistence of *H. pylori* infection.

Infection by *H. pylori* detected preoperatively in 23 of 31 patients resulted unchanged at the 6 months post-operative follow-up. In our series the duodeno-gastric reflux, assessed histologically with the BRI in 58% of the patients after cholecystectomy, seems to have no effect or, at least, no ability to eradicate* H. pylori* from the gastric mucosa.

Although in our series we have not found the presence of intestinal metaplasia of the gastric mucosa in any of the postoperative samples, studies such as the Swedish study [12] are only partially refuted, and associate cholecystectomy with an increased incidence of gastric cancer, potentially attributable to chronic inflammatory insults such as bile reflux gastritis; this condition is all but uncommon in our series of patients who undergo cholecystectomy.

Although these findings remain to be confirmed on a wider coverage, pending further clarification, a clinical and endoscopic follow-up in relation to the suspected potential transformation is recommended in patients who undergo cholecystectomy and in which a chronic bile reflux gastritis is diagnosed. We planned, therefore, to continue our experimental investigation for the next three years, aiming to gather a wider sample of individuals in order to draw further conclusions.

## List of abbreviations

BRI: Bile Reflux Index; DGR: duodenal-gastric reflux.

## Competing interests

The authors declare that they have no competing interests.

## Authors' contributions

FG, AG, AC, AF, AB, MBC, FT, GM:acquisition of data, drafting the manuscript, given final approval of the version to be published, GA,BA: conception and design, interpretation of data, given final approval of the version to be published, FC: acquisition of data, drafting the manuscript, given final approval of the version to be published.
